# Collaborative robots (cobots) for disaster risk resilience: a framework for swarm of snake robots in delivering first aid in emergency situations

**DOI:** 10.3389/frobt.2024.1362294

**Published:** 2024-03-04

**Authors:** Syed Kumayl Raza Moosavi, Muhammad Hamza Zafar, Filippo Sanfilippo

**Affiliations:** ^1^ School of Electrical Engineering and Computer Sciences, National University of Sciences and Technology, Islamabad, Pakistan; ^2^ Department of Engineering Sciences, University of Agder, Grimstaad, Norway; ^3^ Department of Software Engineering, Kaunas University of Technology, Kaunas, Lithuania

**Keywords:** cobots, search and rescue operation, human robot collaboration, snake robots, path planning, disaster scenarios, swarm robots

## Abstract

Cobots are robots that are built for human-robot collaboration (HRC) in a shared environment. In the aftermath of disasters, cobots can cooperate with humans to mitigate risks and increase the possibility of rescuing people in distress. This study examines the resilient and dynamic synergy between a swarm of snake robots, first responders and people to be rescued. The possibility of delivering first aid to potential victims dispersed around a disaster environment is implemented. In the HRC simulation framework presented in this study, the first responder initially deploys a UAV, swarm of snake robots and emergency items. The UAV provides the first responder with the site planimetry, which includes the layout of the area, as well as the precise locations of the individuals in need of rescue and the aiding goods to be delivered. Each individual snake robot in the swarm is then assigned a victim. Subsequently an optimal path is determined by each snake robot using the A* algorithm, to approach and reach its respective target while avoiding obstacles. By using their prehensile capabilities, each snake robot adeptly grasps the aiding object to be dispatched. The snake robots successively arrive at the delivering location near the victim, following their optimal paths, and proceed to release the items. To demonstrate the potential of the framework, several case studies are outlined concerning the execution of operations that combine locomotion, obstacle avoidance, grasping and deploying. The Coppelia-Sim Robotic Simulator is utilised for this framework. The analysis of the motion of the snake robots on the path show highly accurate movement with and without the emergency item. This study is a step towards a holistic semi-autonomous search and rescue operation.

## 1 Introduction

Societies encounter unforeseeable crisis situations such as earthquakes, fires, floods, hazardous spills, hurricanes/typhoons, tsunamis, terrorist attacks, refugee crises, and more. These crises can arise from natural causes or human activities and result in substantial loss of life, injuries, displacement of people, and damage to property. In retrospect, humans have learned to adapt and manage such calamities on a global scale. However, due to an increase in the impact of the ever increase disasters, exacerbated partly due to the climate change, management of such disasters have become complex in this socio-ecological landscape. According to the latest data from insurer Munich Re (Munich, 2023), the average losses due to natural catastrophes over 5 years (2017–2021), adjusted to inflation, was approximately $270bn while the statistics for 2022 alone was over $270bn. Moreover, the impact of disasters are not always recorded in detail when disasters do occur.

### 1.1 Disaster management

The need to manage such disaster situations is apparent. Therefore, it is increasingly important for disaster managers to assume an expanding role in safeguarding their communities through the formulation of effective management strategies. Emergency management processes are commonly categorised into distinct stages, although there is no universally agreed-upon model. On the aftermath of an event, disaster management typically involves four phases (Cova, 1999): to mitigate devastation of effective areas of potential disasters; have preparedness by incorporating trained personnel and shelter facilities; effective response during search and rescue (SAR) operations; recovery during the aftermath of the disaster. Conventional approaches, including field monitoring, physics-based models, expert surveys, and multi-criteria decision-making techniques, are utilised to identify hazards and risk factors. However, these methods often require extensive human effort and involve very high risk. One of the technological tools that can be used in these dangerous environments without adding risk to the life of humans, who are in the process of SAR operation, are robots. By integrating human skills with automation, a harmonious blend can be achieved, leveraging the adaptability of manual processes and the effectiveness and consistency of machines. This synthesis enables the realisation of benefits such as accessing hazardous or hard-to-reach areas, remote operation, adaptability, reducing human fatigue and risk. Particularly, in emergency scenario, there is a need for very close collaboration between first responders and robots. Therefore, collaborative robots, also known as cobots (Jeyson et al., 2022), represent a promising solution.

Many natural and man-made events have occurred in history that have prompted the use of robots for post disaster conditions. In the highly radioactive environment at Chernobyl after the 1986 nuclear blast, the KOMATSU, the TELEROB and many other significant ground vehicles were used for multiple tasks in place of humans (Tochilin et al., 2021; Sparkes, 2022). In 2007, the I-35 bridge collapsed into the fast moving currents, inaccessible to human divers, of the Minnesota river. Remote marine vehicles were then used for search, reconnaissance and mapping underwater (Murphy et al., 2011). In 2012, on the aftermath of the Finale Emilia earthquake in Italy, a combination of unmanned aerial vehicles (UAVs) and unmanned ground vehicles (UGVs) were used for the structural inspection of buildings (Kruijff et al., 2012). The fallout information was provided to the Italian National Fire Corps and state archeologists.

### 1.2 Virtual framework for disaster resilience

To enhance the definition of disaster recovery management strategy, researchers have proposed the integration of collaborative systems in a virtual space (Bertolino and Tanzi, 2019). An example of addressing this issue can be found in a recent study (Magid et al., 2019), where a novel framework and diverse control strategies were introduced for enhancing the collaborative performance of heterogeneous robotic swarms in the context of sensing, monitoring, and mapping flood and landslide disaster zones. The research presents a foundation of virtual simulators that demonstrate various robot interaction protocols and system modelling concepts within the Gazebo environment of the Robot Operating System (ROS). The use of digital twins to enhance human-robot collaboration (HRC) in complex production systems was explored by Malik and Brem (2021), presenting a case study, highlighting the potential advantages, and building blocks of digital twins in the field of collaborative robotics. In Burke et al. (2004), a field study on human-robot interaction during an urban SAR training exercise in Miami was presented, focusing on the challenges and dynamics that arise in this context. In Wagner (2021), an analysis on emergency evacuation as a valuable paradigm for studying human-robot interaction was provided, emphasising the need for ethical considerations. Different design approaches are discussed together with ethical implications, outlines, and a roadmap for the development and evaluation of emergency evacuation robots. In Sanfilippo (2022), the author proposed a simulation case study for SAR operation combining modular robot, grasping, and locomotion capabilities. Thus, computer based virtual models of physical systems can be used to test and validate complex strategies and scenarios prior to their implementation in real world applications.

Building upon virtual models for disaster scenarios, a case study for a SAR simulation environment with HRC using snake robotics is presented in this study. The main contribution of this work includes the development of the control framework for HRC between an unmanned aerial vehicle (UAV), multiple snake robots, a first responder, and multiple victims of a disaster. The proposed architecture distributes the control scheme of the HRC into four phases simulated in a virtual environment: deployment (HRC), path planning and sharing (robot to robot collaboration), grasping and locomotion, releasing first aid item and return. Deployment involves initialising the HRC system by the first responder by positioning the team of snake robots and the UAV; Path Planning and Sharing focuses on optimising collaborative robot paths to prevent collisions; Grasping and Locomotion addresses precise object manipulation and robot movement; Releasing First Aid Item and Return entails placing a first aid item and safely returning to a designated location. The CoppeliaSim - formerly known as Vrep (Rohmer et al., 2013) is adopted as the virtual simulator. Regarding visual perception, a camera is placed at the bottom of the UAV, which is deployed on top of a maze/disaster scenario, for image keypoints extraction, while another camera is attached on the head of the snakes for path following.The respective paths are obtained at runtime by each of the first responders’ snake robot. Multiple models of snake robots (Liljebäck et al., 2014) are used for locomotion on a dynamic path. To prove the efficacy of the design, a case study featuring a maze is presented, which combines human and multi-robot collaboration, locomotion, object grasping and dropping, path following and obstacle avoidance.

### 1.3 Collaborative capabilities of humans and unmanned vehicles in shared spaces

In today’s dynamic and fast-paced world, the integration of unmanned vehicles into shared spaces is becoming increasingly common. Collaborative unmanned systems have emerged to meet our society’s wide-ranging grand challenges, with their advantages including high performance, efficiency, flexibility, and inherent resilience (Zhang et al., 2022). The essence of collaborative capabilities lies in the synergy between humans and unmanned vehicles. It entails a multifaceted approach aimed at harmonising the efforts of both entities to ensure their coexistence in shared spaces. At its core, this collaborative framework is driven by a profound need for seamless and secure interaction, which is underpinned by a range of technological advancements and innovative strategies.

One of the primary challenges that collaborative capabilities address is the navigation of completely unknown dynamic and unstructured environments. These spaces, whether they be urban jungles with constantly changing traffic patterns or uncharted wilderness with rugged terrains, pose significant obstacles. Humans rely on their cognitive abilities and prior experience to navigate these landscapes, but unmanned vehicles depend on a different set of skills. They harness the power of multi-sensor data fusion (Khaleghi et al., 2013), integrating information from various sources such as cameras, LiDAR, radar, and GPS. This fusion not only enhances their perception of the environment but also empowers them to make informed decisions. Through this synergy, unmanned vehicles can navigate through unknown terrains with greater precision, safety, and adaptability.

In the context of disaster environments, enhanced planning and sequential decision-making become even more critical components of collaborative capabilities (Unhelkar et al., 2020). These environments are characterised by extreme unpredictability, chaos, and rapidly changing conditions, making efficient interaction between humans and unmanned vehicles a matter of life and death. In such high-stress scenarios, a proactive approach to planning and decision-making is essential.

Multi-agent coordination (Queralta et al., 2020) is another pivotal component of collaborative capabilities. Shared spaces often involve a multitude of agents, both human and unmanned, operating in tandem. For instance, in a disaster settings, unmanned delivery vehicles may need to coordinate with one another to optimise routes and minimise debris collision, while also interacting seamlessly with victims and other vehicles. Collaborative algorithms, backed by real-time communication and negotiation mechanisms, ensure that these interactions are conducted smoothly and safely. This ability to coordinate with other agents makes shared spaces more efficient and less congested, benefiting both human and unmanned vehicle operators.

Collaborative capabilities extend beyond the realm of autonomous vehicles and encompass human-vehicle interaction. In shared spaces, it is essential that unmanned vehicles communicate with humans in a user-friendly and intuitive manner. Trust and understanding are critical factors in ensuring the success of various applications. Collaborative interfaces may include informative displays, natural language communication, and even gestures to establish a clear and mutual understanding between humans and unmanned vehicles. This level of communication helps build trust and confidence in the technology, ultimately leading to more widespread acceptance.

This paper is organised as follows. A review of the related research work is given successively in Section 2. Then, the model of a snake robot is provided in Section 3. Subsequently, the proposed framework architecture is outlined in Section 4. Simulation results are presented in Section 5. A Discussion is provided on swarm robots and HRC in Section 6. Finally, conclusion and future work are discussed in Section 7.

## 2 Related work

Snake robots have gained significant attention in the field of robotics due to their unique locomotion capabilities and potential applications in disaster scenarios. These robots, inspired by the flexibility and maneuverability of biological snakes, offer a great potential for navigating complex and challenging environments. In rough terrains, steered vehicles often provide non feasible solutions between waypoints due to the kinematic and dynamic limitations, specially on curvatures, even when vehicles can rotate and turn in place (Eskandarian et al., 2019). Snake robots exhibit a range of physical configurations and purposes, although their movement is often inspired by snakes. These robots can differ in terms of redundancy, wheel usage, and even their ability to operate in both land and water environments. Their slender, elongated bodies with thin cross-sections make them particularly well-suited for exploring narrow spaces or pipes. The distribution of mass and the presence of multiple ground contact points contribute to their stability, especially when compared to other robotic designs like wheeled or multipedal systems (Hopkins et al., 2009). Snake robots promise impressive adaptability to various terrains, primarily relying on the roughness of the ground or obstacles to gain sufficient traction and move forward without slipping (Webster et al., 2006). This adaptability and stability in different terrains make them robust to mechanical failure, enabling exploration in uncertain and challenging environments. In terms of gait patterns, snake movement can be categorised into four categories (Seeja et al., 2022): (a) lateral undulation; (b) concertina; (c) rectilinear progression; and (d) side winding.

There are two distinct approaches to snake robot locomotion based on the understanding of the environment: obstacle avoidance locomotion and obstacle accommodation/exploitation locomotion. In cluttered environments, snake robots exploit obstacles as an aid for propulsion purposes. This is known as “obstacle-aid Locomotion” (OAL) (Holden et al., 2014). Snakes utilise a strategy of pushing against unevenness or irregularities in the environment, creating bends in their body. This bending pattern is propagated from the head to the tail, allowing for smoother locomotion. However, this method is heavily reliant on the friction present in the environment, and collisions with obstacles can hinder further motion, potentially causing mechanical stress or damage to the equipment. The significance of environment perception, mapping, and representation cannot be overstated. In fact, our research group has introduced the term “perception-driven obstacle-aided locomotion” (POAL) to underscore this concept. POAL refers to a locomotion approach where a snake robot leverages its sensory-perceptual system to utilise the surrounding operational space. It identifies walls, obstacles, or external objects as means of propulsion. Our group’s work on POAL has been documented in several publications (Sanfilippo et al. (2016a; b, 2017a)). To facilitate the design and simulation of POAL, our research group has developed SnakeSIM, a virtual rapid-prototyping framework. SnakeSIM enables researchers to engage in the safer, faster, and more efficient design and simulation of POAL (Sanfilippo et al. (2017b; 2018)). In terms of control, attaining POAL necessitates precisely identifying possible push-points and properly determining feasible contact response forces. Because of the lack of compliance, achieving this with typical rigidly-actuated robots is exceedingly difficult. To address this challenge, our research group has developed Serpens, a novel modular snake robot equipped with series elastic actuators (SEA). Serpens is notable for its low cost, open-source nature, and high compliance, making it suitable for various applications. We recently introduced Serpens in our research publications (Sanfilippo et al., 2019; Duivon et al., 2022). Regarding guidance, a biologically inspired steering controller was presented in (Rañó et al., 2018). With respect to navigation, a local path planning algorithm for snake robots was introduced in (Hanssen et al., 2020).

Various investigations in literature have concentrated on motion dealing with obstacle avoidance. In this context, the environment perception, mapping, and representation play a crucial role in the overall model. These elements are fundamental for the successful functioning and decision-making of the snake robot in its environment. An example of snake robot motion is the artificial potential field (APF) based locomotion, as described by Davy (2002). This approach involves creating an artificial field around objects, and the robot’s motion is designed to avoid this force field. Another algorithm utilised in snake robot locomotion is the central pattern generator (CPG), mentioned by Nor and Ma (2014). CPG enables the robot to navigate around obstacles or barriers by adjusting the turning of its body from its intended trajectory.

In the field of snake robotics, most of the past literature focuses on specific, static scenarios rather than the possibility of exploring dynamically changing and unpredictable scenarios. This represents a significant research gap. While numerous studies have investigated the locomotion and control mechanisms of snake robots in controlled environments, there is a lack of comprehensive research considering real-world situations where the environment and task requirements dynamically evolve. Such scenarios, which involve navigating through complex and unpredictable terrains, pose unique challenges that need to be addressed to enhance the adaptability and robustness of snake robots. Furthermore, the potential for collaboration between snake robots and other robots or humans is almost untapped. Investigating how snake robots can efficiently work together and engage collaboratively with other entities opens up new opportunities for applications in fields such as SAR, exploration, and HRC. Bridging these research gaps will help to the advancement of snake robot capabilities and their practical deployment in real-world circumstances.

## 3 Modelling

**FIGURE 1:**
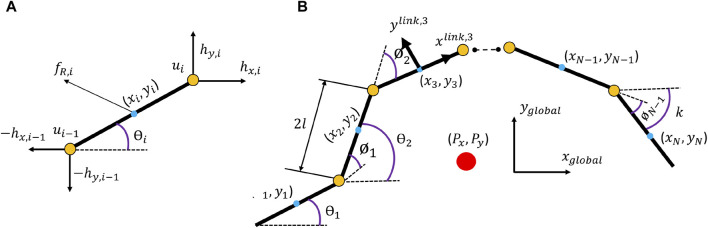
**(A)** Force profile of individual link in a snake robot **(B)** Kinematic and Force/Torque parameters of a snake robot.

To derive a kinematic model for the locomotion of the snake robot on a horizontal and flat surface, Liljebäck et al. (2013) proposed a linearisation of the model due to the many degrees of freedom and the dynamical couplings between links of the robot. A snake robot consists of *N* rigid links of length 2*l* joined by *N* − 1 joints. Each link is assumed to have the same mass *m*, thereby the center of mass of each rigid link is at the center point and the total mass of the snake comes out to be *N* × *m*. The mathematical model is described in terms of the kinematic parameters of the snake robot illustrated in Figure 1B.

The snake robot moves on a horizontal surface with *N* + 2 degrees of freedom. The heading (orientation) of the snake robot is denoted by $\bar{\theta }$ and is defined as the average of link angles as described in Eq. 1.

$$\bar{\theta }=\frac{1}{N}\times \overset{N}{\underset{i=1}{\sum }}\theta_{i}$$ (1)

where link angles (*θ*_*i*_) are defined as the angle that the link forms with the global *x*-axis. On the other hand, joint angles, denoted as *ϕ*_*i*_, is different such that it defines the difference between the link angles between each link i.e., *θ*_*i*_ − *θ*_*i*+1_. The global position of the snake robot is given in eq. 2 as:

$$p=\left[\begin{array}{c} p_{x}\\ p_{y} \end{array}\right]=\left[\begin{array}{c} \frac{1}{Nm}\sum_{i=1}^{N}mx_{i}\\ \frac{1}{Nm}\sum_{i=1}^{N}my_{i} \end{array}\right]$$ (2)

Following this, the forward velocity of the snake robot movement is defined as a component of the center of mass velocity $\left(\dot{p}\right)$ and the current heading as shown in eq. 3.

$${\bar{v}}_{t}={\dot{p}}_{x}\cos\bar{\theta }+{\dot{p}}_{y}\sin\bar{\theta }$$ (3)

Figure 1A shows the joint forces and the friction forces acted upon link *i*. Using the first principle of motion a dynamic model can be described for the whole snake robot in matrix form as shown in eq.4.

$$\begin{array}{cc} m\ddot{X} & =f_{R,x}+D^{T}h_{x}\\ m\ddot{Y} & =f_{R,y}+D^{T}h_{y} \end{array}$$ (4)

where $\ddot{X}=\left[\ddot{x_{i}},\ddot{x_{i+1}}\ldots \ddot{x_{N}}\right]$ , $\ddot{Y}=\left[\ddot{y_{i}},\ddot{y_{i+1}}\ldots \ddot{y_{N}}\right]$ , **f**_*R*,*x*_, **f**_*R*,*y*_ are the ground friction forces. **h**_*x*_ = [*h*_*x*,1_…*h*_*N*,1_] and **h**_*y*_ = [*h*_*y*,1_…*h*_*N*,1_] are defined as the matrix for the joint constraint forces *h*_*x*,*i*_ and *h*_*y*,*i*_ respectively. The torque balance equation for the link *i* is given in eq. 5.

$$\begin{array}{ll} J{\ddot{\theta }}_{i} & =u_{i}-u_{i-1}-l\sin\theta_{i}\left(h_{x,i}+h_{x,i-1}\right)\\ & \;+l\cos\theta_{i}\left(h_{y,i}+h_{y,i-1}\right), \end{array}$$ (5)

where *u*_*i*_ is defined as the torque forces exerted on the link from the next link in the chain of links of the snake robot. By using matrix form and introducing state variables the dynamic model of the snake robot can be compactly described in a state space form as shown in eq. 6.

$$\dot{x}=\left[\begin{array}{c} \dot{\theta }\\ \dot{p}\\ \ddot{\theta }\\ \ddot{p} \end{array}\right]=F\left(x,u\right)$$ (6)

where elements of the **F**(**x**, **u**) can be found in the mathematical breakdown provided by Liljebäck et al. (2013).

## 4 Framework architecture

**FIGURE 2:**
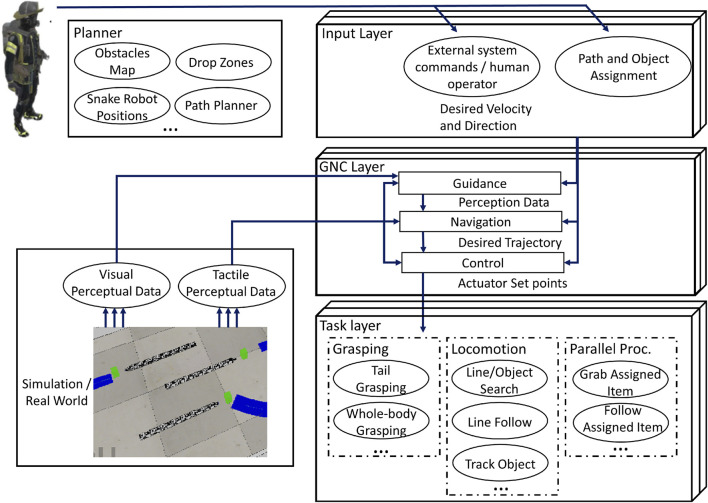
The proposed control framework architecture.

As introduced by Sanfilippo (2022), the selected control framework is organised hierarchically, as shown in Figure 2. The input layer enables the robot to be guided by a human operator to achieve teleoperation or by other external systems (for example, an external planner) to reach higher levels of autonomy.

The core layer is the only layer required to perform the standard functions and capabilities of guidance, navigation, and control (GNC). These characteristics of the unmanned snake robot operate independently of one another and in parallel:

• Guidance: this concerns the process of identifying the desired course or trajectory for the snake robot to follow. It includes the decision-making process that specifies the robot’s objectives and constraints. To generate commands that direct the robot along a desired path, the guidance system considers the robot’s environment, mission requirements, and any other relevant parameters;
• Navigation: it involves the decision-making process regarding the optimal movement of the snake robot, including determining the appropriate location, timing, and method of locomotion. This decision-making process takes into account both external system commands and the sensory data gathered by the snake robot. The desired outcome of the navigation process is to generate a trajectory that includes path and velocity information for the robot to follow;
• Control: it serves as the central component of the presented control framework, offering researchers the flexibility to develop alternative control methods. The inputs to the control module include the desired trajectory and pertinent information obtained from the guidance level, such as perception data. The objective is to determine the necessary setpoints for the robot’s actuators, enabling it to accurately track the desired trajectory.

In this work, a planner is added to the previously presented framework architecture (Sanfilippo, 2022). The planner considers four phases of HRC: 1) deployment (HRC): a first responder deploys the snake robots, a UAV, and first aid items; 2) path planning and sharing (robot to robot collaboration): the UAV captures the planimetry of the area of interest and extracts keypoints of the maze to share with the snake robots. The first responder assigns victims to each snake robot which then form their paths (e.g., shortest path) to their respective targets.

3) grasping and locomotion: the snake robot grasps the first aid item and locomotes to reach the victim to be rescued; 4) releasing first aid item and return: the snake robot delivers and drops the first aid item to the victim to be rescued. Successively, the snake robot returns to the first responder.

The task layer encompasses a variety of tasks that have clearly defined objectives to accomplish. The following tasks have been implemented:

• Line follow: in this task, the robot employs its visual sensor to track a designated line (Kelasidi et al., 2017). Through the utilisation of a proportional integral derivative (PID) controller, the snake robot computes the required adjustments to its locomotion parameters, ensuring that the line remains within the camera’s field of view.
• Line search: in case the vision sensor loses the path following line, for example, during sharp turns, this task performs the operation of rotating and exploring to re-adjust its position to get back on track of the path. The task makes the robot head rotate left and right while making small steps forwards or backwards to find the line.
• Object search: once the Emergency item is detected by the snake robot vision sensor, the line following control scheme is replaced by the object tracking and grasping scheme.
• Locate drop zone: once the drop zone near the victim’s position is detected by the snake robot vision sensor, the line following control scheme is replaced by the object drop sequence.
• Track object: upon detection of an object with a specific color, this particular task employs a PID controller to compute the precise adjustments required for the snake robot’s parameters. These adjustments are aimed at maintaining the object within the camera’s field of view, thus ensuring continuous tracking.
• Pregrasping: in this task, it is assumed that the object to be grasped is positioned in front of the snake robot and is visible to the camera prior to initiating the execution. The snake robot begins by executing a continuous bending motion until the object to be grasped is no longer within the camera’s field of view. Subsequently, a series of forward steps are performed by the snake robot, followed by a continuous bending motion of the head, aiming to detect a collision with the object to be grasped. This sequence is repeated until the object is determined to be in an optimal position for grasping. In the event that the object to be detected does not experience collision, the snake robot moves backwards until the object to grasp is back into the field of view of the vision sensor.
• Grasping: after the pregrasp task is completed, a series of bending maneuvers is then executed to transition the snake robot into the whole-body grasping posture. The snake robot adapts its shape to the object to grasp by determining minimum number of modules (*n*_min_) needed to accomplish grasping according to Eq. 7.

$$n_{\min}=\frac{C_{\mathrm{obj}}}{l_{m}}$$ (7)

where *C*_*obj*_ minimum circular length of object and *l*_*m*_ is the length of the module. To ensure the object remains correctly positioned throughout this sequence, torque sensing is implemented at the joint level. Once the bending procedure concludes, collision detection is employed to verify that the object is correctly positioned within the body of the snake. With the object grasped, the snake robot rotates itself until the path line is in front of the vision sensor, thereby, the line following locomotion begins.

• Drop zone tracking: this task ensures the drop zone is in front of the snake and within the camera’s field of view via PID controller for precise adjustments.
• Dropping: the snake robot advances towards the drop zone, continuing until it is no longer visible within the camera’s field of view. At this point, the snake robot will proceed to move forward for a predetermined duration, based on empirical observations. Following this, the snake robot will come to a stop and release the object by straightening its body, with the center section of the snake pushing the object onto the drop zone.
• Return to line: after the snake robot drops an item, it moves backwards for a predefined number of steps and then performs a sinusoidal rotatory movement to get back on track of the path line.

At the aftermath of a disaster event, first responders arrive at the scene to provide assistance. Due to obstructions from the fallout of the disastrous event, the victims are unreachable by the first responders. In the first phase of the HRC, the rescue responders deploy the snake robots and emergency items (e.g., a water tank or an oxygen tank) in close proximity to themselves, while a UAV is launched on top of the area of interest, which is cluttered with obstacles. Since the environment is dynamic and unknown to the snake robots, assistance from the rescue responder is required. The rescue responder provide the telemetry of the map to their deployed snake robots by the use of a UAV that is equipped with a vision sensor to create a map of the environment. The UAV is responsible for mapping out positions of key elements in the area of interest which include obstacles, positions of multiple victims, first aid items and positions of the snake robots. The control framework for the mapping task procedure of the second phase is itemised as follows:

• Obstacles Map: this task uses the static image taken from the UAV to mark positions of the obstacles in the maze. It also marks the boundary regions creating a bound space of the map for the snake robot traversal.
• Snake robots’ position: this task compares the pre-defined template of a snake robot with every area in the map to determine the position and orientation of the robot. The line path points to be made start from head of the snake robot.
• Drop zone positions: this task determines the position of the drop zone on the maze nearest to the victim. The line following path will end near this position.
• Path planner: once the requisite positions are determined. The snake robots creates path points with small increments on the simulation environment. The path starts from each snake’s position and ends near their drop zone. The control scheme involves A* algorithm (Wang et al., 2015) with the obstacles as the heuristics of the algorithm for optimal path planning.
• Path creation: this task takes the path points and converts them according to the simulation world environment. These points are then embedded on the map maze. A line is then joined between each point to create the path on the maze which the snake robot will follow.

**FIGURE 3:**
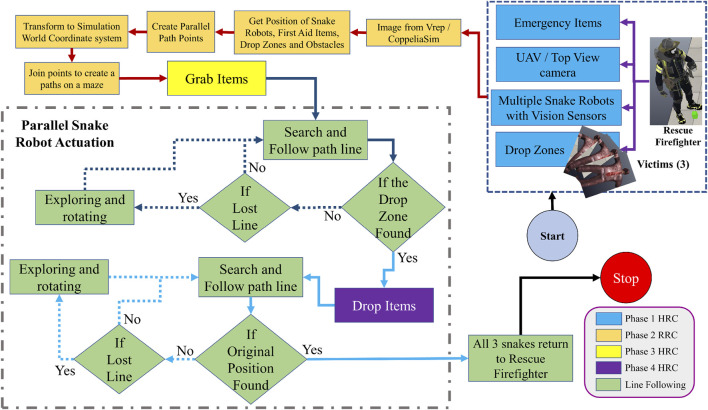
Simulation flowchart.

With this, the system enters the third phase of the HRC process. The snake robot grabs the item from the first responder and positions itself to get on track of the path line. It is important to note that in this work, a swarm of snake robots is considered, operating collaboratively to fulfill the mission objectives. Each snake robot within the swarm contributes to the overall retrieval and delivery process. The robot follows the path line to the position of the victim. When the robot’s vision sensor detects the drop zone near the victim, the fourth phase of the HRC system initiates. In this phase, the robot performs the tasks of dropping the object on the drop zone. After task completion, the snake robots re-adjusts themselves back onto the line and follow the path line back to the first responder, consequently, ending the simulation when the final snake robot has returned. The state machine flowchart of the four-state HRC phases is shown in Figure 3.

## 5 Simulation results

Due to the complexity of the environment, real-world development of control algorithms for snake robots can be challenging. Testing novel control approaches can potentially damage both the environment and the snake robot and can be time consuming. A realistic simulator is much more efficient for the development of control strategy. Coppelia Sim (Rohmer et al., 2013) is chosen as the simulation environment in this study because it is a flexible simulation framework that supports multiple operating systems. Each module can be controlled via embedded script, plugins, a remote application programming interface (API) client or a user-defined solution. Lua lightweight, multi-paradigm programming language (Ierusalimschy et al., 2012) - created in 1993 - is used within the Coppelia Sim simulator.

To demonstrate the potential of the proposed framework for Search and Rescue (SAR) operations, a case study presented in this work offers simulation results. The purpose of the operation is for the multi-snake robot swarm to retrieve a distinctly colored object (marked as a green cube) from a first responder and bring it to the victims in need (at the drop zone). It is crucial to highlight that within this simulation, there are multiple victims requiring assistance, and a number of first responders who interact with the swarm of snake robots.

The simulated autonomous planner for this operation is divided into two main parts, each contributing to the successful accomplishment of this complex objective:

• A remote API - via Jupyter Notebook (Kluyver et al., 2016) - initialises the simulation, performs all the image recognition tasks and determines the path points, using the A* algorithm, from the snake robots to the drop zone positions. These path points are then converted to the simulation cartesian coordinate system and sent to the Coppelia Sim simulator. A child script written embedded in each snake robot model in the simulator receives these points and draws their path onto the maze.
• The snake robots starts their locomotion simultaneously (rectilinear progression) by first performing the pregrasping/grasping operation to get the emergency item and place the robot where the path line is in front of the snake head vision sensor. By avoiding obstacles, the snake robots traverse toward the drop zone following the path line. The snake robot then drops the object onto the drop zone, readjusts itself back to the path line and returns to the original position near the first responder, consequently ending the simulation.

**FIGURE 4:**
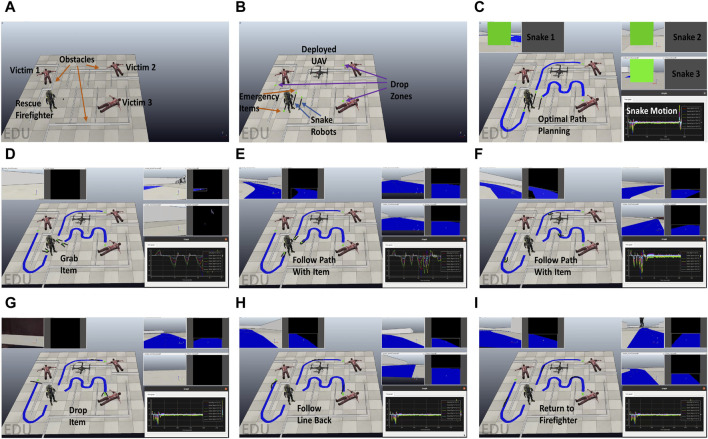
A sequence of successive screenshots for the selected four phase HRC system case study. The screenshots contain the simulated environment, raw and processed video streams of the snake vision sensor and robot joint’s (7 in total) force / torque measurements. **(A)** Disaster Scenario **(B)** HRC Phase 1 **(C)** HRC Phase 2 **(D−F)** HRC Phase 3 **(G−I)** HRC Phase 4.

A sequence of successive screenshots for the selected four phase HRC system case is shown in Figure 4.

## 6 Discussion

**FIGURE 5:**
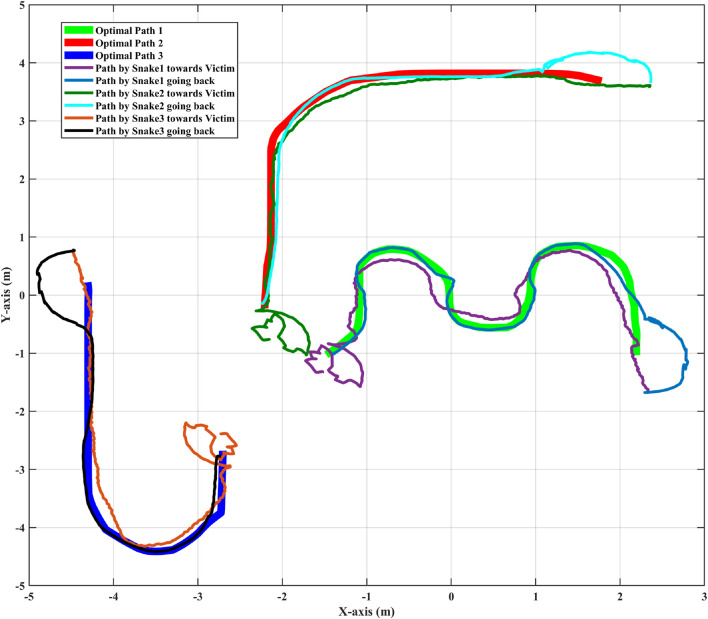
Navigational trajectory of autonomous snake robots and adaptive grasping/ungrasping movements executed upon locating emergency item/victims.

**FIGURE 6:**
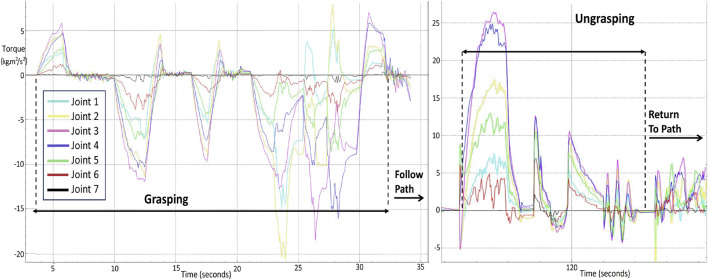
Torque profiles of seven joint actuators in a snake robot (Joint 7 is at the tail of the snake), showing the variations in mechanical stress during grasping, path-following, and ungrasping phases over time.

A disaster scenario, man-made or natural, consists of many unknown variables and dangers. Human intervention becomes difficult if the risk of falling debris is a factor. In such situations a swarm of robots being deployed would be considered as an ideal scenario for SAR operations. Bio-inspired snake robots can traverse a variety of challenging terrains, such as narrow paths, uneven surfaces, as well as gravel, and debris, among others, where robots with different mobility systems may encounter significant difficulties. The snake like structure of the robot aids in locomotion into tight spaces. The collaborative synergy between snake robots and human responders amplifies the impact of SAR efforts, enabling the possibility to reach locations that otherwise might be unapproachable. Furthermore, the modular nature of snake robots allows for customisation and adaptability. Different modules can be attached or detached enhancing their versatility. This modularity aligns with collaborative efforts, as snake robots can be equipped with various sensors, cameras, or tools to aid in data collection, assessment, and interaction with the environment. The path to the victim followed by the snakes, grabbing the emergency item in the process and their subsequent return back following the defined path line, is showcased in Figure 5. The figure illustrates that the slender design of the snake aids in maintaining adherence to the path even with the emergency item in grasp. Figure 6 displays the torque profiles of the snake’s joints during different phases of the HRC process. A maximum torque value of 15 *kgm* ^2^/*s* ^2^ is needed during the grasping process while for ungrasping the joint force of 25 *kgm* ^2^/*s* ^2^ is needed. Joints 3 and 4 require the highest torque during ungrasping because the snake robot has to unclench the item and push it forward towards the victim drop zone. The head of the snake is responsible for turning and rotating to grab the item, therefore, the tail, which is Joint 7, shows the least amount of torque required during the grasping phase.

Snake robots, while innovative and adaptable, come with inherent limitations. Adaptability to terrains with different friction properties is still an open challenge (Chitikena et al., 2024). Energy efficiency poses a concern due to the energy-intensive movement. Additionally, snake robots typically have limited payload capacity. Their slender, elongated design prioritises agility and flexibility over the ability to carry heavy loads. This limitation affects their utility in applications requiring the transport of significant equipment or materials, restricting their roles to surveillance, data collection, and light manipulation tasks. Furthermore, operating these robots requires specialised knowledge and skills, as their movement patterns are complex and vastly different from more conventional robotic systems. Moreover, communication reliability, slower speeds, and ethical considerations (Chitikena et al., 2023) must also be addressed for effective collaboration. Balancing these drawbacks with their benefits necessitates ongoing research and tailored deployment strategies.

The use of multi-snake robots also offers a solution to a common limitation in disaster response: limited human resources. In disaster scenarios, the availability of trained human responders can be scarce or overwhelmed. Snake robots can operate autonomously and require minimal human intervention, thereby alleviating the pressure on the limited pool of responders and allowing them to focus on tasks that require human expertise and decision-making. However, there are several challenges to be addressed in the implementation of a swarm of snake robots in disaster environments. Firstly, ensuring effective communication and coordination among the robots is crucial. The development of robust communication systems that can withstand interference and transmit critical data in real-time is a significant technological challenge. The integration of advanced sensors and perception capabilities is another area of focus. These snake robots must be equipped with state-of-the-art sensors to provide comprehensive situational awareness.

## 7 Conclusion and future work

The adoption of collaborative robots (cobots) by disaster managers heavily relies on their capabilities, reliability, and robustness during field deployments. The extent of autonomy exhibited by robotic systems not only influences the manpower needed for their operation but also determines the complexity and adaptability of the system. However, achieving full autonomy in real-world rescue scenarios is currently challenging and not readily applicable in practical situations. Nevertheless, there is a clear inclination towards incorporating semi-autonomous behaviours instead of relying solely on manual control. This approach aims to alleviate the cognitive burden on the operator, enabling them to multitask or operate multiple systems concurrently. However, it is crucial to involve humans in the decision-making loop to guide the robot’s actions, particularly in tasks that involve dynamic changes throughout the mission, such as search and rescue operations during disaster scenarios. This paper proposes a control framework for human-robot collaboration (HRC) in an environment for disaster scenarios. A simulation using the Coppelia Sim (Rohmer et al., 2013) interface is used, in which a swarm of robots is emulated using an unmanned aerial vehicle (UAV) and multiple snake robots. The swarm of snake robots is deployed to navigate through a dynamic disaster scenario cluttered with obstacles, with the objective of retrieving an emergency item from the first responder, such as an oxygen tank or a water tank, and delivering it to various victims scattered throughout the provided map. The UAV provides data of the site planimetry to the snake robots. Using the A* algorithm the snake robot follows the optimal path towards the victim on the maze. The control framework considers four phases of HRC: deployment (HRC), path planning and sharing (robot to robot collaboration), grasping and locomotion, releasing first aid item and return. Simulation results show the efficacy of devising such a system in a physical environment for the future. Torque plots and navigational trajectory of the autonomous snake robots demonstrate the practicality of implementing such design frameworks in real world scenarios. In future, multiple robots can be assigned distinct roles, such as search and rescue, hazard assessment, structural assessment, or overlapped tasks for a common goal and they can execute these tasks concurrently. Physical obstacles may also be replaced with synthetic fire (in mixed reality) for obstacle avoidance.

## Data Availability

The original contributions presented in the study are included in the article/Supplementary material, further inquiries can be directed to the corresponding author.
